# An experimental assessment of *in silico *haplotype association mapping in laboratory mice

**DOI:** 10.1186/1471-2156-10-81

**Published:** 2009-12-09

**Authors:** Sarah L Burgess-Herbert, Shirng-Wern Tsaih, Ioannis M Stylianou, Kenneth Walsh, Allison J Cox, Beverly Paigen

**Affiliations:** 1 The Jackson Laboratory, 600 Main Street, Bar Harbor, ME 04609, USA; 2 Current address: San Diego Zoo Conservation Research, 15600 San Pasqual Valley Road, Escondido, CA 92027, USA; 3 Current address: University of Pennsylvania, School of Medicine, Institute for Translational Medicine and Therapeutics, Philadelphia, PA 19104, USA

## Abstract

**Background:**

To assess the utility of haplotype association mapping (HAM) as a quantitative trait locus (QTL) discovery tool, we conducted HAM analyses for red blood cell count (RBC) and high density lipoprotein cholesterol (HDL) in mice. We then experimentally tested each HAM QTL using published crosses or new F2 intercrosses guided by the haplotype at the HAM peaks.

**Results:**

The HAM for RBC, using 33 classic inbred lines, revealed 8 QTLs; 2 of these were true positives as shown by published crosses. A HAM-guided (C57BL/6J × CBA/J)F2 intercross we carried out verified 2 more as true positives and 4 as false positives. The HAM for HDL, using 81 strains including recombinant inbred lines and chromosome substitution strains, detected 46 QTLs. Of these, 36 were true positives as shown by published crosses. A HAM-guided (C57BL/6J × A/J)F2 intercross that we carried out verified 2 more as true positives and 8 as false positives. By testing each HAM QTL for RBC and HDL, we demonstrated that 78% of the 54 HAM peaks were true positives and 22% were false positives. Interestingly, all false positives were in significant allelic association with one or more real QTL.

**Conclusion:**

Because type I errors (false positives) can be detected experimentally, we conclude that HAM is useful for QTL detection and narrowing. We advocate the powerful and economical combined approach demonstrated here: the use of HAM for QTL discovery, followed by mitigation of the false positive problem by testing the HAM-predicted QTLs with small HAM-guided experimental crosses.

## Background

Model organisms facilitate the discovery of genes through classical experimental methodologies and, more recently, through the application of bioinformatics resources and tools. Inbred line crosses have been successfully uncovering quantitative trait loci (QTLs) of complex traits since the early 1990's [see review in [1]]. Meanwhile, vast improvements in high-throughput methodologies and the resulting availability of genomic data such as genome-wide sequence coverage, single nucleotide polymorphism (SNP) information, and tissue-specific expression data have enhanced the capabilities of classical model organism research. The molecular dissection of complex traits is aided by harnessing these increasingly accessible data through the integration of bioinformatics into traditional methods of gene discovery [2-4].

*In silico *QTL mapping, or as we prefer to call it, haplotype association mapping (HAM), has been proposed as a means of utilizing phenotypic data from inbred strains together with dense marker maps to determine associations of haplotypes with phenotypes [5,6]. Initially this approach was highly criticized on the one hand as having too many statistical issues to be useful, such as a high rate of false positives that could lead to much dead end work [7], while on the other hand it was welcomed with excessive zeal as the end to tedious and expensive experimental crosses [5].

Subsequent reports have tempered these two 2 perspectives somewhat with investigations of the power of HAM when variables and algorithms are altered, such as the number of strains, the "population structure" of the strain panel, the density of markers, the size of the haplotypes, the haplotype inference method, and efforts to control family-wise error [6,8-11]. In addition, some researchers have found HAM useful as part of an integrative fine-mapping method for narrowing known QTLs [12,13] or as a method for QTL discovery [14,15], while others have found that the method lacks power for their phenotype of interest [16].

Understanding the conditions under which HAM succeeds or fails to identify QTLs and the mechanisms by which type I errors (false positives) and type II errors (false negatives) are generated will help researchers wisely use this powerful tool. The number and the genetic relatedness of the inbred strains used in HAM analyses are known to affect its statistical power [8,9,17,18]. In addition, it has been noted that spurious associations among both linked (*cis*) and unlinked markers (*trans*) are likely to be a problem in association studies such as HAM [7,10,12].

In this study, we evaluated the performance of HAM as a tool for discovering and mapping QTLs. We used it to predict QTLs for red blood cell count (RBC) and for plasma concentration of high density lipoprotein cholesterol (HDL) in mice. We then tested whether each HAM-predicted QTL was a real QTL, as determined by a previously reported QTL cross that fit the haplotype at the HAM-predicted QTL peak or by a new cross carried out specifically to test these HAM-predicted QTLs. Our results, which allowed us to calculate the rate of false positives, show that HAM can be a powerful, although not comprehensive, predictor of QTLs even for a highly polygenic trait such as HDL cholesterol. We also show that extensive allelic association, the matching of SNPs at true HAM peaks with SNPs elsewhere in the genome, is responsible for type I error control problems; and we demonstrate a method for mitigating this issue that involves conducting relatively small-scale experimental crosses to test HAM QTL peaks.

## Results

### RBC - red blood cell count

#### HAM - Haplotype association mapping

**Figure 1:**
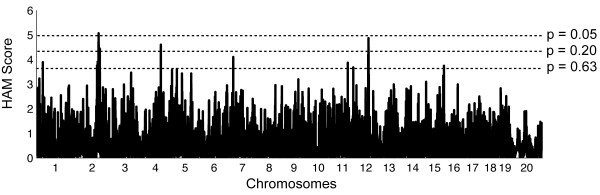
**HAM genome scan for RBC in 33 inbred lines of mice**. X-axis = position in genome, chromosomes labeled. Y-axis = uncorrected HAM score, which is -log10(*P *value). Dotted lines indicate thresholds of genome-wide significance; HAM scores are 4.97, 4.34, and 3.64 for *P *= 0.05, 0.20, and 0.63.

With the trait of red blood cell count, our goal was to assess the usefulness of HAM in discovering new QTLs when working on a quantitative trait for which little was known about its genetic architecture. Haplotype association mapping of 33 classic inbred strains for RBC in females (Figure 1) yielded one significant peak on chromosome (Chr) 2 and 7 suggestive peaks on Chrs 1, 4, 7, 11, 12, and 15 (see Table 1). Here, we defined a HAM "peak" as the individual SNP plus the consecutive 2 SNPs that make up its 3-SNP window (see Methods). Because each unique QTL may be surrounded by SNPs that also were above the level of significance, we have included a column in Table 1 that shows the range of SNPs surrounding the HAM peak that are below the suggestive significance of 0.63. For some QTL, such on the one on Chr 1, only the single SNP window reached suggestive significance, but for the QTL on Chr 2, this range is so large (116-131 Mb) that we suspect a second QTL gene may exist in the region.

**Table 1:** RBC HAM QTL peaks

Chr	SNP ID	Mb	Range	HAM	Bootstrap	*P*
1	rs13475796	32,907,323	-	3.89	73.4%	P < 0.63
2	rs6257970	**124,821,501**	116,276,771-131,106,484	**5.10**	**94.8%**	**P < 0.05**
4	rs3664701	**105,717,060**	105,385,114-105,717,060	**4.60**	**88.5%**	**P < 0.20**
7	rs13479152	24,436,399	24,248,444-24,436,399	4.10	75.9%	P < 0.63
11	rs13481145	88,251,886	-	3.87	79.2%	P < 0.63
11	rs3024036	116,489,336	116,489,336-116,486,660	3.68	65.5%	P < 0.63
12	rs13481527	**73,806,035**	72,236,752-74,576,810	**4.92**	**96.2%**	**P < 0.20**
15	rs3023430	100,626,402	-	3.74	68.5%	P < 0.63

HAM QTL information for significant (*P *< 0.05), highly suggestive (*P *< 0.20), and suggestive (*P *< 0.63) RBC peaks at HAM scores of 4.97, 4.34, and 3.64 respectively. HAM score is -log *P *value. Positions are based on NCBI Mouse Build 36 position (bp) of 1st SNP in 3-SNP window. The range is the locations of first and last SNP surrounding the peak that had a value <0.63. Boldface highlights peaks with *P *< 0.20 significance level.

#### (B6 × CBA)F2 intercross

The next question is which of these 8 HAM-detected peaks detected at a significance of 0.63 represents a true peak and which are false positives. To answer that question, we first examined the literature for red blood cell QTLs and found that 2 of these QTLs on Chrs 4 and 11 have been detected previously with classic inbred line crosses and the haplotype at the HAM peaks differed for the parental strains that detected the QTL [19]; thus these 2 are true positives. To determine whether the remaining 6 peaks are true or false positives, we tested them with a small haplotype-based intercross. At each of the 6 peaks, we determined the haplotypes, examined which pair of strains would be expected to have a QTL peak at that location, and then carried out a cross between CBA and C57BL/6J (B6) because those 2 strains would be able to detect all 6 QTLs if they were true positives. Because we were only testing these 6 locations, we used a small cross of 108 mice and only tested polymorphic markers at or flanking each of the 6 peaks to determine whether the polymorphisms were associated with a difference in phenotype.

**Figure 2:**
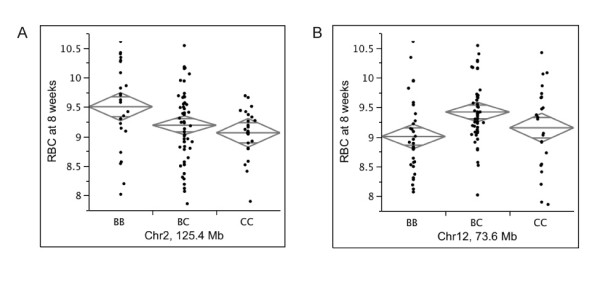
**Significant one-way ANOVAs for RBC in (CBA × B6)F2 females**. RBC measured as number of cells per microliter (n/uL). BB are B6 homozoygotes, BC are heterozygotes, and CC are CBA homozygotes. A. Chr 2 ANOVA at 125.4 Mb (marker SNP = rs6324984, *P *= 0.029). B. Chr 12 ANOVA at 73.6 Mb (marker SNP = rs3714720, *P *= 0.0075).

One-way ANOVAs conducted at markers near, including, or flanking the 6 HAM peaks revealed that the significant peak on Chr 2 (124.8 Mb) and the suggestive peak on Chr 12 (73.8 Mb) were both significantly correlated with RBC (Figure 2). The remaining 4 peaks tested (Chr 1: 32.9 Mb, Chr 7: 24.4 Mb, Chr 11: 116.5 Mb, and Chr 15: 100.6 Mb) were not significantly correlated with RBC (data not shown), so we classified these as "false positives" (Table 2). If one uses a suggestive level of 0.63, this gives a false positive rate of 50% (4 of 8); if we had used a more stringent suggestive level of 0.20, there would have been no false positives for any of the 3 loci detected at this level. However, one true positive on Chr 11 would have been lost.

**Table 2:** Testing whether the RBC HAM peaks are true or false positives

Chr	Mb	True/false	Evidence for QTL	Reference
1	32.9	False	None in CBAxB6	This report
2	124.8	True	QTL in CBAxB6	This report
4	107.7	True	QTL in NZWxSM	19
7	24.4	False	None in CBAxB6	This report
11	88.2	True	QTL in NZWxSM	19
11	116.4	False	None in CBAxB6	This report
12	73.8	True	QTL in CBAxB6	This report
15	100.6	False	None in CBAxB6	This report

#### Allelic association analysis

**Figure 3:**
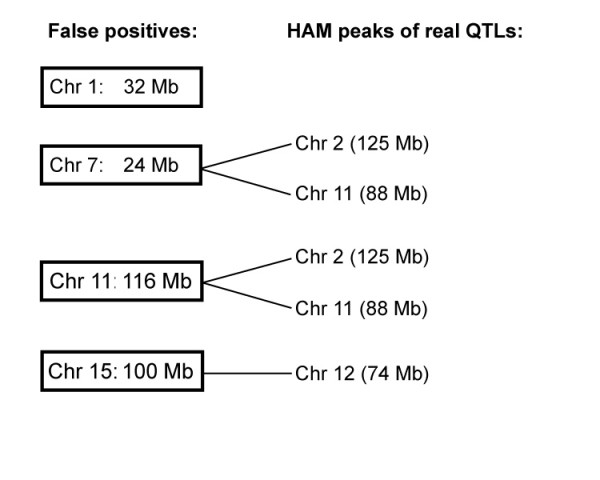
**Allelic association among RBC HAM peaks**. Allelic association among false positive RBC HAM peaks (on left) and true RBC HAM QTLs (on right).

We then tested each false positive locus to determine if it was in allelic association with the experimentally verified HAM QTLs. We used the common statistical test for linkage disequilbrium, which is the same as allelic association except that linkage disequilbrium is restricted to loci on the same chromosome. We tested the non-independence of the false positive HAM peaks by examining the pairwise allelic association among all of the peaks for RBC (Figure 3). All "clusters" of SNPs within a chromosomal peak were found to be in significant linkage disequilbrium with each other, including the cluster of spread out peaks from 116.3 Mb to 131.3 Mb on Chr 2. In addition, the false positives on Chrs 7, 11, and 15 were in significant allelic association with at least one real QTL (Figure 3). The Chr 7 and Chr 11 false positive peaks were in allelic association with both the novel HAM QTL on Chr 2 and with the HAM QTL within the known QTL on Chr 11 [19] detected by a (NZW × SM)F2 intercross. The Chr 15 false positive was in allelic association with the HAM QTL on Chr 12. Only the Chr 1 false positive was not in significant allelic association with any other HAM peaks after a conservative Bonferroni correction for multiple tests.

### HDL - high density lipoprotein cholesterol

#### HAM - haplotype association mapping

**Figure 4:**
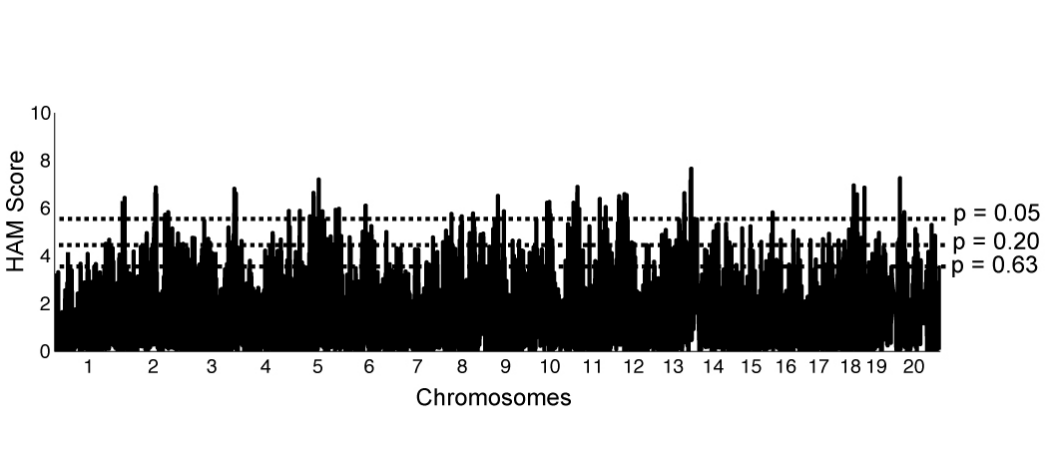
**HAM genome scan for HDL in 81 strains of inbred mice**. X-axis = position in genome, chromosomes labeled. Y-axis = uncorrected HAM score, which is -log10(*P *value). Dotted lines indicate thresholds of genome-wide significance: HAM scores are 5.48, 4.46, and 3.48  for *P *= 0.05, 0.20, and 0.63

Using the trait of HDL cholesterol, our goal was to assess the usefulness of HAM for a quantitative trait with highly complex genetics. At least 40 unique QTLs are known to influence HDL cholesterol levels in mice as estimated from the 111 HDL QTLs identified by over 24 different inbred line crosses [20,21]. Because no peaks reached the genome-wide significance threshold when we performed HAM for HDL with only the 33 classic inbred strains (data not shown), we added the chromosome substitution strains derived from strains B6 and A/J and 2 sets of recombinant inbred lines (AXB, BXD) for a total of 81 strains (see Methods). While this expanded strain panel increased the power of peak detection (Figure 4), it also brought with it additional potential for false positives due to the enrichment of the B6, A/J, and DBA/2 genomes contributed by the consomic and recombinant inbred lines. This HAM yielded 46 peaks significant at the 0.05 level (Table 3). The range in Table 3 represents any SNPs surrounding the peak that also had a significance level <0.05.

**Table 3:** Significant HDL HAM QTL peaks (*P *< 0.05)

Chr	SNP ID	Mb	Range	HAM	bootstrap	True/false	Ref
2	rs6220817	3,011,010	-	6.30	76.2%	False	a
2	rs13476342	8,836,883	-	6.43	75.8%	False	a
2	rs13476342	99,935,969	98,155,571-100,911,329	6.58	77.4%	True	20,21
2	rs13476760	126,832,982	126,336,525-126,832,982	5.74	65.6%	True	20,21
2	rs13476760	135,828,607	-	5.84	63.0%	True	20,21
3	rs13477475	147,010,573	-	6.03	62.4%	False	a
3	rs13477475	151,010,779	-	6.62	77.8%	False	a
4	rs3693400	146,890,343	-	5.89	69.2%	True	20,21
5	rs3693400	55,019,679	54,974,352-55,019,679	5.67	58.2%	True	20,21
5	rs6265085	65,029,889	65,029,889-65,818,061	6.64	80.5%	True	20,21
5	rs6265085	74,887,607	-	5.57	61.3%	True	20,21
5	rs13478349	80,970,712	80,945,292-80,970,712	7.21	87.4%	True	20,21
5	rs13478349	84,064,324	-	5.85	60.8%	True	20,21
5	rs13460234	91,625,499	-	5.87	66.6%	True	20,21
5	rs13460234	97,622,792	-	5.49	57.1%	True	20,21
5	rs3668978	130,797,661	-	5.94	60.6%	True	20,21
5	rs3668978	140,694,495	139,748,376-140,870,443	5.98	67.5%	True	20,21
6	rs13478802	66,337,104	65,537,646-66,337,104	6.11	70.0%	True	20,21
8	rs13478802	36,795,544	-	5.77	61.6%	True	a
8	rs13479806	68,181,411	-	5.67	62.4%	True	a
8	rs13479806	100,597,759	-	5.78	68.0%	True	b
8	rs13479951	103,466,389	-	5.50	62.7%	True	b
9	rs13479951	40,786,256	40,599,802-41,263,933	5.67	70.7%	True	20,21
9	rs4227694	58,060,520	-	5.67	62.8%	True	20,21
10	rs4227694	28,727,018	-	5.54	58.2%	False	a
10	rs13480621	59,831,809	-	6.23	76.1%	False	a
10	rs13480621	66,633,531	66,622,596-69,957,648	6.26	74.1%	False	a
11	rs13480851	7,106,276	7,106,276-7,133,854	6.23	67.3%	True	20,21
11	rs13480851	18,664,287	18,012,274-18,776,572	6.90	76.8%	True	20,21
11	rs13480915	24,340,180	-	5.97	64.7%	True	20,21
11	rs13480915	84,210,490	84,186,523-84,210,490	6.39	71.2%	True	20,21
11	rs13481191	100,981,257	-	6.05	66.5%	True	20,21
12	rs13481191	17,813,143	-	6.51	77.8%	True	20,21
12	rs3655333	29,314,512	29,245,634-29,342,877	6.26	70.0%	True	20,21
12	gnf12.033.545	35,706,448	-	5.78	65.2%	True	20,21
12	mCV23299449	40,033,960	-	6.60	73.7%	True	20,21
12	rs6197363	46,205,386	46,195,413-46,672,638	6.55	67.3%	True	20,21
13	rs6197363	82,973,404	-	6.64	83.5%	True	20,21
13	rs13481961	98,370,936	97,388,131-98,516,414	5.52	60.6%	True	20,21
13	rs13482032	118,459,784	116,568,685-118,459,784	7.66	93.9%	False	a
14	rs6291247	11,729,249	11,724,710-11,804,687	5.53	56.7%	True	20,21
14	rs3678171	16,445,566	16,421,809-16,469,365	5.53	56.7%	True	20,21
16	rs4163564	13.779,788	-	5.83	62.7%	True	b
18	rs13483379	57,799,450	-	6.96	76.3%	True	20,21
18	rs3663770	67,730,210	67,730,210-67,748,515	6.59	72.4%	True	20,21
18	rs13483493	89,985,557	-	6.87	79.6%	True	b

a this report

b unpublished crosses B6 × NOD and B6 × NZW by Paigen et al.

HAM QTL information for significant (*P *< 0.05) HDL peaks at HAM scores of 5.48. Positions are based on NCBI Mouse Build 36 position (bp) of 1st SNP in 3-SNP window. The range is the locations of first and last SNP surrounding the peak that had a value <0.05.

#### Testing for true positive peaks

As shown in Table 3, 36 of these 46 HAM peaks were true positives because they were detected previously in inbred line crosses [20,21] and because the haplotype at the HAM peaks differed for the parental strains that detected the QTL. To test whether the remaining 10 peaks were true or false positives, we tested them with a haplotype-based intercross as we did for RBC. At each of the 10 peaks, we determined the haplotypes, examined which pair of strains would be expected to have a QTL peak at that location, and then carried out a cross between B6 and A/J because those 2 strains would be able to detect all 10 QTLs if they were true positives. We tested polymorphic markers at only those 10 peaks to determine if a true QTL existed.

**Figure 5:**
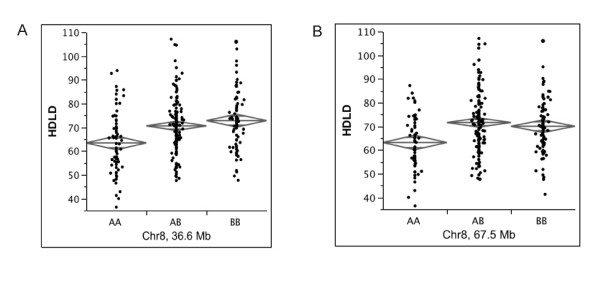
**Significant one-way ANOVAs for HDL in (B6 × A)F2 males**. HDLD is concentration of plasma HDL (mg/dL). AA are A homozygotes, AB are heterozygotes, and BB are B6 homozygotes. A. Chr 8 ANOVA at 36.6 Mb (marker = D8Mit191, *P *< 0.0001). B. Chr 8 ANOVA at 67.5 Mb (marker = D8Mit25, *P *< 0.0001).

One-way ANOVAs revealed that 2 peaks (Chr 8: 36 Mb and Chr 8: 68 Mb) are significantly correlated with HDL (Figure 5). The other 8 peaks (Chr 2: 3 and 8 Mb; Chr 3: 147 and 151; Chr 10: 28, 59, and 66 Mb; Chr 13: 118 Mb) failed to show a phenotype-genotype correlation (data not shown), confirming that they are indeed HAM false positives, yielding a 17% false positive rate for this trait under these mapping conditions.

#### False negatives

**Figure 6:**
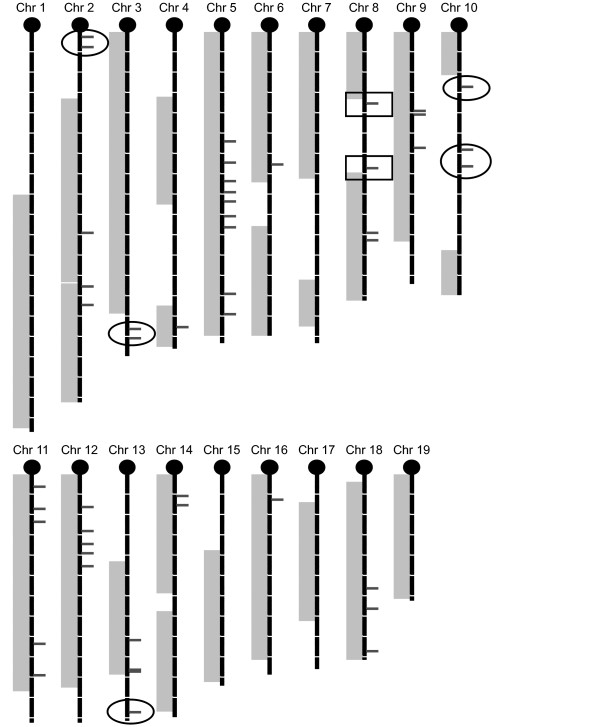
**Chromosome map of HDL HAM peaks and known HDL QTLs**. Black chromosome sections = 10 Mb. Marks to right of chromosomes denote HAM peaks. Gray areas to left of chromosomes indicate regions corresponding to the combined 95% confidence intervals of all known HDL QTLs. Ovals are drawn around false positive HAM peaks; boxes (Chr 8) are drawn around HAM peaks outside of known HDL QTLs that tested positive in the (B × A)F2 test cross.

Since most inbred line crosses conducted now re-find HDL QTLs mapped previously by other crosses, we feel that the QTL map for HDL is almost saturated. This allows us to ask the question of false negatives -- whether HAM misses QTLs found by crosses. To answer that question, we mapped the 95% confidence intervals of HDL QTLs for each inbred line cross onto a physical map and indicated the peak locations of the 46 HAM peaks (Figure 6). This figure has boxes around the 2 new QTL peaks on Chr 8 detected first by HAM and then confirmed by the intercross described above. It also has ovals around the false positives on Chr 2, 3, 10, and 13. The gray bars indicate known QTLs as determined by crosses; several of these have no HAM peaks (Chrs 1, 3, 4, distal 6, 7, 10, distal 14, 17 and 19) indicating a number of false negatives or QTLs that were not detected by the HAM analysis. Calculating a false negative rate is uncertain because some of these regions may contain more than one QTL. However, there are at least 12 QTL regions that were not detected by HAM. If we add these 12 to the 38 true positives, that provides a total of 50 QTLs; since 12 were missed by HAM, the lowest estimate of a false negataive rate is 24% (12/50).

#### Allelic association analysis

**Figure 7:**
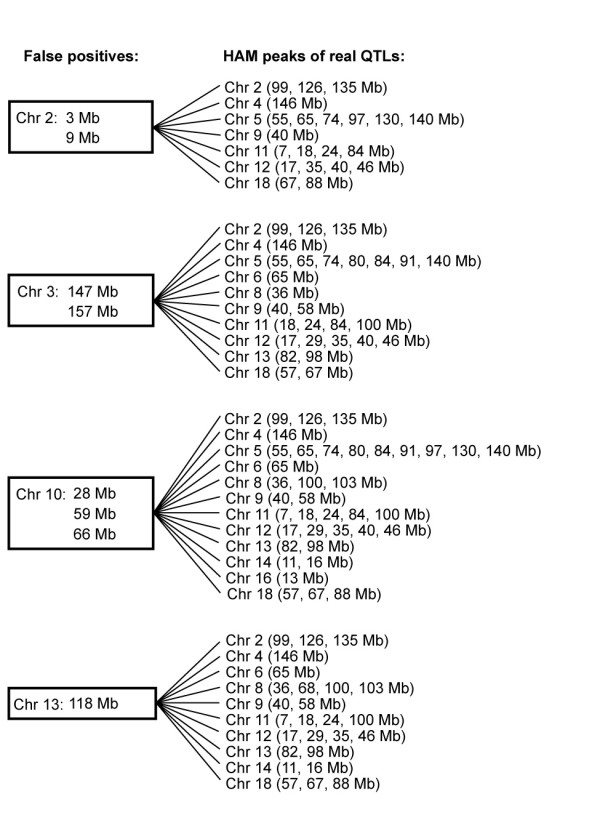
**Allelic association among HDL HAM peaks**. Allelic association among false positive HDL HAM peaks (on left) and true HDL HAM QTLs (on right).

We tested each of the 8 false positive loci for HDL to determine if each was in allelic association with experimentally verified HAM peaks. After testing for pairwise correlations, we found that all false positives were in allelic association with real HAM QTLs on multiple chromosomes (Figure 7).

## Discussion

Haplotype association mapping was developed as a tool for quantitative trait analysis. Initially conceived as an inexpensive and rapid alternative to intercrosses for finding QTLs, limitations of statistical power and type I error control have restricted its widespread adoption. Whereas other studies have suggested that HAM be included in an approach to dissecting complex traits that involves haplotype association mapping within known QTLs [12,22], we demonstrate here the additional proficiency of HAM in detecting novel QTLs. We show that using genome-wide HAM to predict QTLs and then testing the novel QTLs by small haplotype-guided intercrosses can successfully pinpoint QTLs, even for highly complex traits like HDL cholesterol. We also prove the role of allelic association in HAM's pervasive false positive problem in laboratory mice, an issue mitigated by our method of testing HAM-predicted QTLs in small crosses. Detecting QTLs by HAM is a balance between reducing missed QTLs (false negatives, or type II errors) while increasing false positives (type I errors), and increasing missed QTLs while reducing false positives. However, because one well-chosen and relatively small cross can test many HAM QTL peaks using only targeted genotyping, we feel that it is better to err on the side of reducing the missed QTLs while increasing the false positives.

Overall, combining the results of RBC and HDL, we found 12 of 54 HAM peaks were false positives. This may be an underestimate since some of the "true positives" confirmed by previous crosses may not actually be true. We think this particularly likely if 2 HAM peaks are close together such as those on distal Chr 8 or proximal Chr 14. Only identification of the QTL genes will show whether such closely linked QTL peaks are both real. We estimate a false positive rate of 24%, a number that is almost certainly an underestimate and highly dependent on the number of strains used.

Using HAM, even quantitative traits with completely unknown or little-known genetic architecture can be economically explored. An example is the exploration of red blood cell count reported here. Using one HAM-directed small-scale experimental cross with targeted genotyping, we discovered 2 novel RBC QTLs, including a QTL on Chr 2 that includes a strong candidate gene for red blood cell count. The parental strains of the RBC test cross were based solely on their haplotype differences at the RBC HAM peaks and, in fact, are not very different in terms of mean RBC count (units per volume × 10^6 ^[n/uL]) with the mean RBC for B6 at 9.186 n/uL and the mean RBC for CBA at 9.232 n/uL. Upon testing the F2 progeny from this cross, we confirmed the presence of RBC QTLs on Chrs 2 and 12, which represent QTLs not yet found by traditional crosses. Furthermore, a cursory examination of the genes underlying the Chr 2 HAM QTL reveals a likely candidate gene, erythrocyte protein band 4.2 (*Epb4.2*). EPB42 is a major component of the erythrocyte membrane skeletal network that regulates the stability and flexibility of erythrocytes, and is implicated in the maintenance of normal surface area and cation transport in red blood cells [23-25]. As further evidence that it is the likely quantitative trait gene underlying this novel QTL for red blood cell count, in humans and mice, a deficiency of EPB42 results in hemolytic anemias of varying severity [26].

Our results agree with the report of lung tumor susceptibility phenotypes [22], which used HAM to find QTL peaks and then used previously published crosses to determine whether the peaks were true or false. Our report goes somewhat further in using the haplotypes at the HAM-predicted peaks to carry out a small cross designed to test each of the peaks not previously found. Since we conducted the HAM analysis and then carried out the crosses to test novel peaks, a number of advances that will improve HAM and reduce the false positives have occurred. The density of the SNP maps and the number of strains for which SNPs are available are improving rapidly. Moreover, improved algorithms that account for admixture and population structure will help minimize false positives, such as the efficient-mixed model association (EMMA) method of Kang et al. [18]. For example, we think that the high number of false positives for HDL were due to the enrichment of B6, DBA/2, and A genomes contributed by the consomic and recombinant inbred lines, and that the EMMA method of controlling for strain relatedness would have reduced these. In addition, the fact that all but one of the false positives in our experiments were in allelic association with a true QTL peak suggests that the 3-SNP window is too restrictive and that using a HAM algorithm with a haplotype-based window might be a better approach. We subsequently tried HAM using a haplotype based on a Hidden Markov Model and imputed SNPs [27] and found that the percentage of false positives was reduced. However, since the crosses were already completed, we reported the results we obtained with our initial analysis using a 3-SNP window. We also expect that the Collaborative Cross [28] will further improve the resolution of HAM in laboratory mice by disrupting some of the pervasive linkage disequilibrium found in the mouse genome. As a starting point for HAM analyses, researchers can download and upload phenotype information on inbred lines from and to online resources such as the Mouse Phenome Database http://www.jax.org/phenome[29,30].

## Conclusion

In sum, although HAM is not likely to detect all QTLs contributing to a complex trait and is insufficient as a replacement for traditional QTL mapping, we conclude that the integrated method presented here is a powerful addition to our toolbox for quantitative trait analysis. Instead of conducting large scale expensive crosses, HAM can help find new QTLs, which can then be verified using smaller crosses designed to test specific regions of the genome. Additionally, since such crosses are chosen based on their haplotypes, this process can lead to the discovery of QTLs that may not be found by the standard method of basing parental strain choice on highly divergent phenotypes.

## Methods

### Animal housing & blood collection

Mice were obtained from The Jackson Laboratory and housed under conditions meeting the guidelines issued by the Association for Assessment and Accreditation of Laboratory Animal Care. All animal protocols were approved by The Jackson Laboratory's Animal Care and Use Committee. Mice were housed in 31 × 31 × 214 cm polycarbonate cages that were divided into 2 pens with no more than 5 mice/pen (Thoren Caging Systems Inc.) in rooms maintained at a 12:12 light:dark cycle and an ambient temperature of 23°C. Each cage was pressurized and individually ventilated (PIV) with HEPA filtered air supply and exhaust. Mouse colonies were regularly monitored for 15 viruses, 17 bacterial species (including *Helicobacter *spp., *Pasteurella pneumotropica*, and 2 *Mycoplasma *spp.), ecto- and endo- parasites, and the microsporidium *Encephalitozoon cuniculi *http://jaxmice.jax.org/health[31].

All mice were fed a regular chow diet (6% fat, 5K52 from LabDiets^®^) and water *ad libitum*. About 200 uL of blood for hematological analysis was obtained from 8-10 week old mice in both the HAM strain panel and the (B6 × CBA)F2 intercross by retro-orbital bleeding. For lipid analysis, plasma was separated from blood by centrifugation.

### Phenotyping

Red blood cell count was measured using a Bayer ADVIA^® ^120 Hematology Analyzer from Siemens Diagnostics. A SYNCHRON^® ^CX5 Delta Clinical System chemistry analyzer from Beckman Coulter was used to measure the lipid profiles of mouse plasma. The concentrations of high density lipoprotein cholesterol from these profiles were quantitatively determined using the SYNCHRON^® ^Systems Lipid Calibrator (HDLD assay).

### Haplotype association mapping

The sex specific lipid and hematopoietic traits of inbred lines of mice, including the B.A chromosome substitution strains and the BxA and BxD recombinant inbred lines (RILs), were measured. Using 4-15 mice per strain, we calculated the sex specific trait means for HDL and RBC (http://www.jax.org/phenome[30]; datasets "Stylianou and Paigen" and "Peters1/MPD6202" respectively). HDL values were log_10 _transformed. These trait data were input into our analysis as vectors. A SNP panel consisting of over 21,000 autosomal SNPs was compiled from SNP resources including Wellcome Trust, Broad Institute, and Perlegen, and missing SNPs were inferred from adjacent SNPs (complete SNP panels with RBC and HDL trait strain means and HAM results are available at http://cgd.jax.org/datasets/phenotype.shtml[32]. For RBC, we included 33 classic inbred strains in our strain panel; for HDL, we included 32 classic inbred strains and added the 20 B.A chromosome substitution strains, 15 BxA RILs, and 14 RILs, for a total of 81 strains. These strain panels were input into our analyses as genotype matrices.

We computed regression-based test statistics to measure the strength of association between 3-SNP haplotypes and averaged mean phenotypes to detect haplotype groupings with significantly different mean phenotypes. Since the segregation of strains into genotypic groups varies widely over haplotype blocks, *P *values rather than the test statistics were reported and used for comparisons among haplotype blocks. All *P *values were transformed using -log_10_(*P *value) to produce HAM scores for the scan plots. We controlled type I error rate for multiple testing due to genome-wide searching using family-wise error rate control [33]. Permutations were performed by shuffling the phenotype data while keeping the genotype data intact. The minimum *P *value was recorded for each permutation, and percentiles of their distribution were used to provide approximate multiple test-adjusted thresholds. The genome-wide type I error thresholds were estimated based on 1000 permutation tests. 3-SNP windows, or "peaks" corresponding to *P *value thresholds adjusted for global significance were defined as follows: *P *< 0.05 as significant, *P *< 0.2 as highly suggestive, and *P *< 0.63 as suggestive [34]. We estimated "peak stability" by calculating bootstrap values per peak based on 1000 bootstraps. All analyses were done in the MATLAB computing environment (The Mathworks, http://www.mathworks.com[35].

### F2 intercrosses

We examined the strain haplotypes at the significant and suggestive peaks and chose strains for intercrosses based on which strains had different haplotypes. Phenotype differences between strains were not considered. For RBC, the C57BL/6J (B6) and CBA/J (CBA) haplotypes differed at all of the 6 relevant peaks in females, so we set up a small F2 intercross of B6 females crossed with CBA males. The resulting F2 females (N = 108) were phenotyped for RBC at 8 weeks old. For HDL, the B6 and A/J (A) haplotypes differed at all 10 significant peaks that were not confirmed by published HDL QTLs. Because our lab had already conducted a large (B × A)F2 intercross for kidney disease [36], we used the plasma stored at -80°C to measure HDL and used the saved DNA from 292 males for genotyping. (When these crosses were decided upon, phenotyped, and tested at the HAM peaks of interest for this study, the Chr 8 HDL QTLs were discovered here and were novel. However, while this paper was in preparation, the same (B × A)F2 cross DNA was subsequently genotyped at additional locations across the genome and was published by Stylianou *et al*. [37] as a traditional genome-wide QTL analysis in conjunction with an evaluation of HDL QTLs in B.A chromosome substitution strains.)

### Genotyping

Genomic DNA was extracted from the tail-tips of the F2 progeny using a standard phenol-choloform procedure. SNPs flanking, within, or nearby HAM peaks of interest were genotyped by the Allele Typing Service at The Jackson Laboratory in conjunction with KBiosciences Ltd. In 6 cases, microsatellite markers were used instead: D1Mit373, D2Mit1, D8Mit191, D8Mit25, D11Mit48, and D15Mit149. For these, genomic DNA was amplified in PCR reactions with 0.200 mM of each dNTP, 0.132 uM of each MIT marker primer, 1× AmpliTaq buffer, and 0.375 Units of AmpliTaq^® ^DNA Polymerase (Applied Biosystems) under the following amplification conditions: initial denaturation at 95°C for 5 minutes, 35 cycles of 95°C (30 seconds) + 55°C (30 seconds) + 72°C (1 minute), and a final elongation at 72°C for 7 minutes. PCR products were visualized on 2% agarose gels and scored by eye.

### Statistical analyses

Intercross progeny were genotyped at markers flanking, including, or nearby the HAM peak of interest. Using JMP^® ^by SAS, we tested each marker for correlations between phenotype and genotype with one-way ANOVAs. We tested the non-independence of the HAM peaks using an analysis of "linkage disequilibrium" in GENEPOP, http://genepop.curtin.edu.au[38,39]. Pairwise comparisons of the 3-SNP haplotypes at the HAM peaks for each trait were conducted by contingency table analysis using a Markov chain and G-based probability tests as implemented in GENEPOP, with 1000 permutations. A Bonferroni correction was applied to the results in each case to account for multiple testing. For RBC, all HAM peaks above the suggestive *P *< 0.63 threshold were tested for the non-random association of their haplotypes with each other, while for HDL, all significant HAM peaks above the significant *P *< 0.05 threshold were tested for the non-random association of their haplotypes with each other.

## Authors' contributions

SLBH designed the experiments, carried out statistical analyses for peak testing and linkage disequilibrium, and wrote the manuscript. S-WT set up and conducted all final HAM analyses, carried out peak stability bootstrapping, participated in SNP panel construction, and guided initial HAM analyses. IMS planned and coordinated phenotyping, participated in SNP panel construction, and participated in initial HAM analyses. KW performed all genotyping. AC conducted RBC and HDL phenotyping and assisted with intercross experiments and manuscript revisions. BP conceived of the study, participated in its design and coordination, and helped write the manuscript. All authors read and approved the final manuscript.
